# Psychosocial issues and sleep quality among seafarers: a mixed methods study

**DOI:** 10.1186/s12889-022-13154-4

**Published:** 2022-04-09

**Authors:** Fereshteh Baygi, Farzad Shidfar, Ali Sheidaei, Aliasghar Farshad, Morteza Mansourian, Christine Blome

**Affiliations:** 1 Research Unit of General Practice, Department of Public Health, University of Southern Denmark, Odense, Denmark; 2 Occupational Health Research Centre, Iran University of Medical Sciences, Tehran, Iran; 3 Department of Nutrition, School of Public Health, Iran University of Medical Sciences, Tehran, Iran; 4 Department of Epidemiology and Biostatistics, Tehran University of Medical Sciences, Tehran, Iran; 5 Health Promotion Research Center, Iran University of Medical Sciences, Tehran, Iran; 6 Institute for Health Services Research in Dermatology and Nursing (IVDP), University Medical Center Hamburg-Eppendorf (UKE), Hamburg, Germany

**Keywords:** Work life at sea, Seafarers, Stress, Mixed methods study, Sleep quality

## Abstract

**Background:**

Seafarers, especially those working for a prolonged period of time, are exposed to a high number of social, psychological and physical stressors including irregular working hours.

**Aim:**

This study aims to identify important aspects of Asian seafarers’ psychosocial wellbeing and quality of sleep that could affect safety and health of the seafarers in long voyage tankers.

**Methods:**

In this mixed method study, psychological health issues were investigated through semi-structured interviews with 17 Asian male seafarers. Participants were selected through purposive sampling. In the quantitative phase, 179 seafarers completed the Pittsburgh Sleep Quality Index (PSQI) on sleep quality.

**Results:**

From the interviews, three categories consisting of six themes emerged, including sleep abnormalities, inevitable stressors, and lack of social communication. The majority of the seafarers believed that their quality of sleep was affected by their physical work environment and by issues raised by their families. As reasons for stress at sea, they mentioned the nature of the occupation and the psychosocial work environment. Most participants pointed out that lack of social communications had adverse effects on both their work lives on board and their private lives at home. In the quantitative phase, the response rate was 81.4%. Mean PSQI index was 5.9 (SD 2.6 and range from 2 to 14). Sleep impairment was higher in academic than non-academic individuals (*p* < 0.001).

**Conclusion:**

Sleep abnormalities and lack of social communication should be considered as modifiable risk factors for seafarers’ psychosocial well-being at sea.

## Background

Seafarers work and live in a working place with specific requirements regarding safety and health. This work environment puts high demands on seafarers compared to those working on land [1]. On long voyage tankers, working tasks are characterized by a wide range of activities such as watch keeping, cargo load and discharge under time-pressure and frequently during the night which are stressful by themselves [2]. Seafaring is therefore associated with relevant mental health risk [3].

Long voyage sailing, irregular working hours and navigation in harsh weather conditions are some of the difficulties of working on board of large vessels. Seafarers’ well-being can be affected by a number of such chronic stressors [4]. Other challenges of working at sea include the inability to leave the workplace, living and working in the same environment, and restricted contact with family members for a long time [2, 5–7]. Moreover, seafarers of tankers operate in a closed community isolated from the shore-based life for at least 4–6 months.

Irregular working hours can disturb sleep and lead to some psychological problems in the long run [8]. Besides, working in rotating time frame is known as a risk factor for metabolic syndrome, oxidative stress, and some of other non-communicable diseases risk factors [9]. Several studies on seafarers’ physical health status have revealed that the prevalence of these risk factors such as metabolic syndrome is high among seafarers [10, 11]. While there is broad literature on adverse psychological effects of working conditions on mental and overall health of workers in land-based occupations [12, 13], much less is known about how this applies to seafarers. Limited studies have been done on this topic [14–16]. Furthermore, the emphasis of most of these studies is on fatigue and burnout of seafarers but not on psychological wellbeing in general [16–18].

Middle Eastern countries – especially Iran – are the main oil and gas producing and exporting countries in the world, and seafaring is a crucial occupation on such countries. According to the studies, working conditions on ships affect the health of seafarers and consequently also the safety of the ship [16, 17, 19, 20]. Workplace environment in Iran has provided an appropriate atmosphere to develop spiritual growth [21]. Such spiritual environment may not only enhance psychosocial wellbeing of employees but also is beneficial for better work performance and safety of the vessel [22]. To date, we know of no study about psychosocial challenges and sleep quality of Asian seafarers. Since the workplace in such countries is characterized by a specific spiritual cultural context [22], the above-mentioned studies may not capture the full picture of psychosocial wellbeing of Asian seafarers. This mixed methods study aims to provide a deep understanding on the topic of sleep quality and well-being of seafarers working on oil tankers. This may improve the knowledge necessary for health promotion interventions that mitigate the adverse effects of working environment on seafarers of middle eastern countries.

### Description of the setting

The current study was done in one of the shipping companies that operate very large crude carrier tankers (VLCC). Such tankers are designed to carry and transform crude oil to oil terminals or platforms. Average crew size in such multinational tankers is about 30–35 individuals.

Seafarers’ ranks on such vessels are as follows: 1) Captain: top-level manager on board; he is responsible for safety of the ship and all crew members. 2) Officers: 1st officer is the second commander of the ship and in charge of cargo operation, deck maintenance, and on-board training of the crew; 2nd officer is in charge of navigation; 3rd officers are responsible for navigation, maintenance of life saving, and firefighting appliances. 3) Engineers: chief engineer is the first commander of engine room; 2nd engineers are responsible for maintenance of all machinery; 3rd engineers along with 4th engineers are responsible for maintenance of some machinery of engine room, also junior engineers assist the 4th engineers. 4) Bosun is the head of deck crew (sailors). 5) Pump man is an experienced seaman during cargo operation and is responsible for maintenance of deck machinery. 6) Able seamen are engaged with operating deck gear and standing anchor details. 7) Ordinary seaman -the lowest ranking in the deck department- the main tasks are lookout and cleaning of the vessel. 8) Engine fitter, oiler, and wiper. 9) Kitchen staff: procurement officer, chief cook, the first cook, the 2nd cook, and messman.

## Materials and methods

### Study design

This mixed method study was conducted in the framework of the need assessment phase of a PhD thesis aiming at implementation of health promotion interventions among Asian male seafarers working in long voyage tankers. All ships investigated in this study were oil tankers of similar size (dead weight: 330,000 tones, length: 333 m, breadth: 60 m) and sailing in international routes. The primary research questions were as follows:

RQ1: What organizational, family, and community elements can influence the psychosocial wellbeing of seafarers on VLCC tankers from their own perspective?

RQ2: What are the experiences of seafarers about psychosocial working environment and other influencing factors on their psychosocial well-being on VLCC tankers?

RQ3: How is the quality of sleep among seafarers who are working on VLCC tankers and which factors is it associated with?

### Qualitative phase

#### Data collection and participants

For the qualitative part of the study, semi-structured in-depth interviews were conducted with seafarers of one of the vessels in the period of 6 months from May to October 2016. To achieve better understanding about working and living conditions at sea – which will improve the quality of qualitative analysis – the main researcher who was native Persian speaker stayed onboard during the mentioned period and all interviews were done on board. An interview guide was developed by research team members according to the primary research questions.

The participants were selected by maximum variation purposive sampling, [23] continued until data saturation. Saturation is reached when no new themes can be found in the interviews [24]. Participants were male seafarers > 18 years with different ranks and job categories on board of one of the multinational VLCC tankers. Having at least 6 months of sea service before joining this vessel and being fluent in Persian language were inclusion criteria. Maximum diversity of information was achieved by including staff members from the three following groups: 1) deck professionals: captain, duty officers, and sailors 2) engine workers: chief engineer, electrician, duty officers, and engine sailors 3) kitchen staff: chief cook, cooks, and waiters.

Before each interview, the purpose of the study was explained. Written informed consent for interviewing and audio recording were obtained from those willing to participate. Individual in-depth interviews were conducted in a private relaxed environment. Additional questions could be asked during the interview in response to the participant’s statements to achieve comprehensive answers to the research questions. The interviewer made field notes for the key points mentioned in the interview and on participants’ body language. Each interview lasted up to 1 h (ranging 35–60 min). All interviews were transcribed verbatim.

#### Coding and analysis

Data collection was done by the main researcher on board of one of the sailing vessels. Analysis was done by two researchers separately to ensure data consistency.

We used MAXQDA 2010 software for data management and coding [25]. Data coding was done in three ways: open, axial, and selective. The first author designed a coding scheme using open coding [26]; results were shared with the second researcher, who revised and added new concepts. The relationship between open codes was identified by axial coding. Then, by selective coding, one core variable was defined that was applicable to all data assigned to this code. Throughout all coding stages, authors discussed with each other, and codes were revised accordingly.

### Quantitative part

The Persian version of the Pittsburgh Sleep Quality Index (PSQI) on sleep quality [27] was completed by 179 seafarers. Inclusion criteria were the same as for the qualitative sub-study.

### Sample size

In shift workers, the prevalence of poor sleep quality has been estimated at 20.4% [28]; we used this number to compute the sample size. According to the formula below for sample size calculation for prevalence of characteristics among the population [29], setting alpha at 0.05 and marginal error (d) equal to 0.1, a sample size of 63 persons was needed.

$$n=\frac{z^2P\left(1-P\right)}{d^2}$$

As almost all demographic and explanatory variables we used were binary, we multiplied by two the sample size. We also increased it by 50% to cover the effect of missing and non-response error, resulting in a minimum required sample size of 189.

On average, 35 seafarers work on one ship. In order to reach the above minimum sample size, the questionnaire was sent randomly to six sailing vessels by email (220 seafarers).

### Data collection

PSQI was used to assess the seafarers’ sleep quality over the previous month. PSQI is a widely used self-rated sleep questionnaire, validated in different populations [27, 30]. From its 19 items, seven component scores are computed, each ranging from 0 to 3. A global score ranging from 0 to 21 is built by summing up the component scores, with higher scores indicating worse sleep quality [31].

### Statistical analysis

Quantitative data were analyzed using Stata version 11.1 (Stata Corporation, College Station, TX, USA). Continuous variables are expressed as mean and standard deviation and categorical variables as frequency and percentage.

The PSQI was also analyzed on a single-item basis. The 5th item describes sleep complaints during the last month using nine sub-items (excluding other reasons for sleep complaints). We compared the frequency of these items with marital status (married/not married), shift work (yes/no), and educational level (academic vs. non- academic) and the PSQI items 1 to 4 (sleep quality, latency, duration and efficiency). The first three of these variables are binary variables; therefore, the two-sample Wilcoxon rank-sum (Mann-Whitney) test was used. The last four items are ordinal and were compared by ‘nptrend’ command, which performs the nonparametric test for trend across ordered groups developed by Cuzick (1985) [32], which is an extension of the Wilcoxon rank-sum test. Finally, we categorized the PSQI score according to the cut-off value 5. The odds ratios of being in severe condition (more than 5) were calculated for each risk factor.

## Results

### Qualitative phase

Data saturation occurred after interviewing with 17 male seafarers. Table 1 shows the summary of participant information in both the qualitative and the quantitative part of the study. Age range was 25–55 years; job history ranged 2–24 years. Most respondents were married, and almost half had academic education. Of the 17 participants, seven, six, and four were from the deck, engine, and kitchen, respectively.

**Table 1:** Characteristic of the studied population

	Participants	Educational level	Marital status	Age	Job history (year)
**Qualitative part**	**P**_**1**_	Master degree	Married	42	17
	**P**_**2**_	Bachelor degree	Married	37	15
	**P**_**3**_	Bachelor degree	Single	37	15
	**P**_**4**_	Bachelor degree	Married	26	9
	**P**_**5**_	Bachelor degree	Single	37	15
	**P**_**6**_**,**	Bachelor degree	Married	35	12
	**P**_**7**_	Bachelor degree	Married	29	4
	**P**_**8**_	Diploma	Married	35	15
	**P**_**9**_	High school	Married	55	24
	**P**_**10**_	High school	Married	44	15
	**P**_**11**_	Diploma	Single	25	2
	**P**_**12**_	High school	Married	55	25
	**P**_**13**_**,**	High school	Single	25	2
	**P**_**14**_	High school	Married	28	4
	**P**_**15**_	High school	Single	26	3
	**P**_**16**_	Diploma	Married	39	17
	**P**_**17**_	Bachelor degree	Married	33	8
**Quantitative part**	**Age**	Mean, SD	36.17, 9.54		
	**Education**	High school	5 (2.79)		
		Diploma	50 (27.9)		
		Bachelor degree	75 (41.9)		
		Master degree	16 (8.94)		
		Lower than high school	33 (18.44)		
	**Marital status**	Single	43 (25.6)		
		Married	125 (74.4)		
	**Shift type**	Day work	52 (29.05)		
		Shift work	127 (70.95)		

**Fig. 1:**
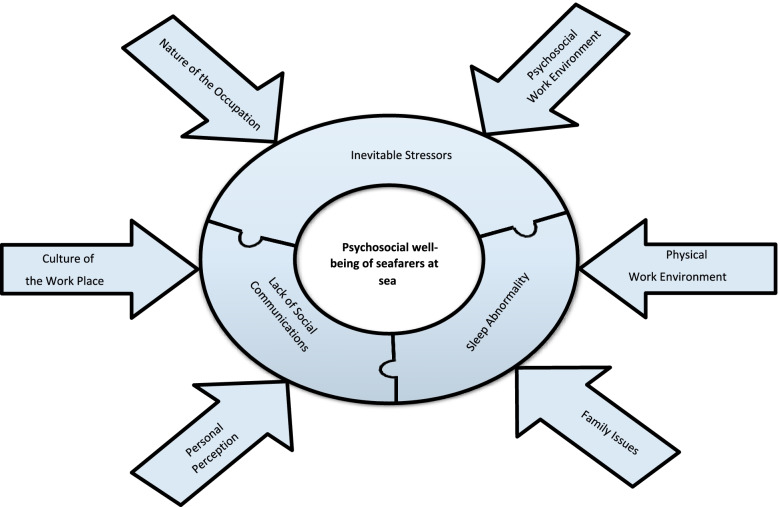
Open coding results on psychosocial aspects of seafarers’ living and working conditions on board

In the qualitative analysis of psychological findings, we found three categories describing factors that may influence psychosocial well-being: sleep disorder, inevitable stressors, and lack of social communication (Fig. 1). Each of these categories included two sub-themes (thus, six in total).

### Sleep abnormalities

All seafarers indicated that sleep abnormalities are a common issue while they are on board. The majority mentioned that work environment and workplace conditions can have adverse effects on their sleep habits and quality. In this respect, P_9_ stated that “on the ship, ventilation has a significant impact on our sleep. Imagine you had a bad dream about your family, and you wake up soaked with sweat and the cabin is also so hot. You won’t be able to stand it and you have to go to take a shower, so your sleep will be over”. Another example provided by P_9_ was: “Here, you should get used to lack of sleep. During operation (load and discharge), we have to stay awake for two or three nights depending on cargo load/discharge rate”.

Sleep quality was also connected with the fact that seafarers had limited contact with their families, mostly via internet during load or unload in different ports. At sea, they did not have internet. Most of them believed that separation from family members without any communication is emotionally demanding. For example, one respondent (P_2_) said that “being away from home and family causes depression. When you want to go to bed at night, you think of your home and family and start to worry about them. When this thought comes to your head, you cannot get any sleep at night”.

As additional reasons for sleep abnormalities, participants mentioned: ship’s movement in harsh weather conditions (rolling); the noise caused by the elevator and the phone in the cabin during rest time; raising responsibilities at upper level and key officers; heavy workloads in high temperature during operations; hearing negative news about the family.

### Inevitable stressors

The category “Inevitable stressors” includes the two sub-themes *nature of occupation* and *psychosocial work environment*. Most participants mentioned that people who work on land may also face different levels of stress, but that reasons for stress on board are completely different in comparison to the land-based jobs. They also believed that such stress perception is basically because of the particular tasks they have on board (e.g., port state control inspection, vetting inspection). An example was given by P_2_: “As an international job, we typically have a routine check-up. We have a vetting any time we dock to a port, which is stressful. We need to prepare ourselves to go through the vetting successfully”.

P_3_ said that: “You have no holidays on the ship and this stresses you out”. Another participant (P_7_) mentioned that “I do not think there is any job like this on the land where you work for 3 months, 4 months, or 6 months without a day off “.

The maltreatment of crew members by high-ranking officers, an aspect of the psychosocial work environment, was mentioned by some participants. An example was given by P_8_: “On some ships, the newly arrived waiter is afraid to deliver his service. As some of high rank people are pretty strict. That stresses the waiter”.

Other aspects mentioned in this category were not having a colleague in the same job category as oneself and the minor role of a sailor in the family especially in making decision.

### Lack of social communication

Most participants pointed out that lack of social communication had numerous adverse effects on their private lives at home as well as their work lives on board of the ships. Personal perception and culture of the workplace were the two themes which emerged in this category.

Seafarers reported to feel contradiction in personal perception about their role in private life because of the deficit in communication with their family and friends. Most participants felt that after coming back home they were like strangers for a couple of weeks. For example, P_5_ stated: “First, when we go home, we are out of the home atmosphere for a while. We totally feel like strangers. There may have been a series of incidents that we do not know anything about, especially when we spend longer time at the sea”.

P_1_ mentioned:” The types of encounters are different due to the relationships we have on the ship. When we go home, we are in a defensive mode for a while. We do not talk to see what others are saying so we can adapt. After all this, our 3 months off is over and we have to go back on the ship”.

Participants even believed that the disciplined culture of the workplace at sea might influence their personality in the long run. Participant 9 pointed out that “we are not used to land life. On the land, my wife sleeps until 10 o’clock in the morning and that is bothering me. We are used to a strict routine system, and they are used to another system. This system is not compatible with the land system”.

Further aspects mentioned in this category: forced and repetitive relationships with other employees; order-centered relationships with lower ranking personnel and occupational hierarchy; isolation and reduced interaction with others after promotion; the impact of the ship’s atmosphere on the relationships between individuals; being harassed by other family members’ behavior at land; changing the type of socializing (isolation and confinement).

#### Quantitative part

From 220 participants, 179 filled out and returned the questionnaires (response rate of 81.4%). The characteristics of the population are shown in Table 1. The mean age of the participants was 36.2 years; 125 individuals (74.4%) were married and 127 (71.0%) were shift workers. The most frequent educational degrees were bachelor’s degree (41.9%) and diploma (25.7%). No differences in demographic and quantitative data were observed between the 6 ships.

Table 2 shows the PSQI results. The mean PSQI index was 5.92 (SD = 2.59); for academic and non-academic individuals, it was 6.52 and 4.64, respectively (*p* < 0.001). Looking at the scores for the different questionnaire components, we found that sleep duration and sleep efficiency differed by shift work. Shift workers fall sleep later than day workers. Additionally, the efficiency of their sleeps was lower. Sleep latency and sleep disturbance were different across educational levels. So that, individuals with academic education suffer from more disturbance and fall asleep later than others. Marital status was not associated with sleep components. The individuals with academic educational level were 4.17 (1.97–8.85) more likely to confront the sleep issue than the other seafarers.

**Table 2:** Quality of sleep measured by PSQI scores in the studied population

Components	Total Mean (SD)	Shift work Mean (SD)		Education Mean (SD)		Marital status Mean (SD)	
		Day work	Shift work	Non- academic	Academic	Single	Married
Comp.1: Sleep quality	0.23 (0.50)	0.25 (0.44)	0.22 (0.05)	0.24 (0.56)	0.24 (0.50)	0.32 (0.71)	0.21 (0.41)
Comp.2: Sleep latency	1.39 (0.91)	1.3 (0.93)	1.43 (0.90)	0.98 (0.77) ^a^	1.51 (0.92) ^a^	1.26 (0.93)	1.42 (0.90)
Comp.3: Sleep duration	1.14 (0.85)	0.92 (0.62) ^a^	1.23 (0.92) ^a^	0.73 (0.53) ^a^	1.36 (0.95) ^a^	1.09 (0.83)	1.15 (0.85)
Comp.4: Sleep efficiency	0.48 (1.01)	0.25 (0.74) ^a^	0.57 (1.09) ^a^	0.10 (0.30) ^a^	0.71 (1.21) ^a^	0.39 (0.90)	0.50 (1.04)
Comp.5: Sleep disturbance	0.96 (0.57)	1 (0.56)	0.94 (0.58)	0.74 (0.56) ^a^	1.05 (0.53) ^a^	0.84 (0.65)	0.98 (0.54)
Comp.6: Use of sleep medication	0.03 (0.17)	0.06 (0.24)	0.02 (0.13)	0.06 (0.24)	0.02 (0.15)	0.00 (0.00)	0.04 (0.20)
Comp.7: Daytime dysfunction	1.68 (0.54)	1.63 (0.48)	1.70 (0.57)	1.68 (0.58)	1.66 (0.56)	1.79 (0.51)	1.64 (0.56)
Global PSQI Score	5.92 (2.59)	5.32 (1.90)	6.17 (2.80)	4.64 (1.65) ^a^	6.52 (2.85) ^a^	5.70 (2.80)	5.96 (2.56)
OR (Global PSQI Score more than 5)		1	1.15 (0.57–2.32)	1	4.17 (1.97–8.85)	1	1.81 (0.88–3.72)
Component1: #9 Score Component2: #2 Score (≤15 min = 0; 16–30 min = 1; 31–60 min = 2, > 60 min = 3) + #5a Score (if sum is equal 0 = 0; 1–2 = 1; 3–4 = 2; 5–6 = 3) Component3: #4 Score (> 7 = 0; 6–7 = 1; 5–6 = 2; < 5 = 3) Component4: (total # of hours asleep)/ (total # of hours in bed) × 100 > 85% = 0, 75–84% = 1, 65–74% = 2, < 65% = 3 Component5: Sum of Scores #5b to #5j (0 = 0; 1–9 = 1; 10–18 = 2; 19–27 = 3) Component6: #6 Score Component7: #7 Score + #8 Score (0 = 0; 1–2 = 1; 3–4 = 2; 5–6 = 3)							

^a^Difference is statistically significant at level of 0.05 according to two samples t-test

The analysis of the individual sleep complaints (PSQI question 5 with 9 sub-items) (Table 3) showed that most complaints were association with higher educational level. Furthermore, married individuals were more likely to wake up in the middle of the night or early morning (*p* = 0.003). Shift working was associated with having pain while sleeping (*p* = 0.05). Finally, individuals with more sleep complaints also fell asleep more lately and slept more (*p* < 0.05). The most prevalent sleep complaint was waking up in the middle of the night or early morning (68.57%), followed by getting to sleep late (66.67%).

**Table 3:** Association between sleep complaints (PSQI question 5 including 9 sub-items), demographic variables and sleep schedule (PSQI items 1–4) (z (*p* values))

	Marital status^b^ (Single/Married)	Shift work^b^ (day work/shift work)	Education^b^ (not academic/academic)	Q1 ^c^	Q2^c^	Q3 ^c^	Q4 ^c^
Cannot get to sleep within 30 min	− 1.15 (0.25)	− 1.77 (0.08)	−3.99(< 0.001^a^)	− 0.14 (0.89)	4.55(< 0.001^a^)	− 0.68 (0.49)	−5.8(< 0.001^a^)
Wake up in the middle of the night or early morning	−2.95 (0.003^a^)	0.85 (0.40)	−2.56 (0.01^a^)	0.18 (0.86)	2.48 (0.01^a^)	−1.72 (0.09^a^)	−2.42 (0.02^a^)
Have to get up to use the bathroom	0.93 (0.35)	0.20 (0.84)	−0.34 (0.73)	−4.6(< 0.001^a^)	2.43 (0.02^a^)	− 0.69 (0.49)	−1.61 (0.11)
Cannot breathe comfortably	−1.87 (0.06)	0.04 (0.96)	−2.32 (0.02^a^)	−2.2 (0.03^a^)	2.74 (0.01^a^)	0.42 (0.67)	−1.30 (0.19)
Cough or snore loudly	−0.53 (0.60)	0.14 (0.89)	−1.79 (0.07)	−1.08 (0.28)	1.59 (0.11)	−0.78 (0.43)	−1.50 (0.13)
Feel too cold	−1.86 (0.06)	−0.53 (0.59)	−1.31 (0.19)	−2.66 (0.01^a^)	0.86 (0.39)	1.33 (0.18)	−2.19 (0.03^a^)
Feel too hot	−1.64 (0.10)	0.56 (0.57)	−2.68 (0.01^a^)	1.09 (0.58)	2.89 (0.004^a^)	0.66 (0.51)	−2.16 (0.03^a^)
Have bad dreams	−1.28 (0.20)	0.77 (0.44)	−1.98 (0.04^a^)	−2.02 (0.04^a^)	1.43 (0.15)	0.43 (0.67)	−0.73 (0.47)
Have pain	1.60 (0.11)	1.92 (0.05^a^)	−0.40 (0.69)	0.83 (0.41)	1.23 (0.22)	0.86 (0.39)	−0.98 (0.33)

^a^Statistically significant at level 0.05 of type 1 error

^b^Two-sample Wilcoxon rank-sum (Mann-Whitney) test. Negative z statistics reflects higher frequency for the second group of column variable

^c^Cuzick test. Negative z statistics reflects negative association or higher values in column variable occur rarely in row variable

Q1: When have you usually gone to bed?

Q2: How long (in minutes) has it taken you to fall asleep each night?

Q3: When have you usually gotten up in the morning?

Q4: How many hours of actual sleep do you get at night?

## Discussion

In this mixed method study, psychosocial work risk factors were explored qualitatively, and for the first time, sleep quality of Asian seafarers working on oil tankers was assessed quantitatively using a standardized instrument.

In the light of previous findings [33], it was surprising that fatigue was not mentioned by any of our participants.

### Sleep

Qualitative results revealed that seafarers regard sleep quality as one of the major health impairments they experience onboard. In the quantitative study, PSQI scores were significantly higher among seafarers with academic educational level. This finding was supported by qualitative results as some of the interviewees mentioned growing responsibilities at upper level and for key officers as additional reason for sleep abnormalities: Also, the association of having pain while sleeping with shift working was supported by qualitative results: participants pointed out the irregular load and discharge as one of the reasons for sleep difficulties.

Poor sleep quality has been associated with absenteeism and occupational accidents in workers with long working hours or rotating work hours; participants with insomnia symptoms has a twofold risk for occupational accidents [34]. Sleep disorders were also mentioned by the majority of our participants as one of the main problems at sea; this may also lead to negative work outcomes like non-efficient performance on board – which means that the safety of the vessel would be at risk. It is therefore in the interest of shipping companies to help improve the quality of their employees’ sleep by providing facilities like good communication means or job trainings to manage stress.

According to the study conducted on seafarers of ocean-going vessels, sleep efficiency of participants was low in both day workers and watch keepers, and average sleep periods were shorter, especially in watch keepers [35]. We found similar results on sleep efficiency and duration of shift workers and day workers.

In US navy personnel, factors negatively affecting sleep duration were work duration and hours of sunlight exposure [36]. According to the circadian timing system, daytime sleep is out of phase with the diurnal rhythm. So, more sunlight exposure will reduce sleep duration it can also cause more awakening and low performance [37]. Such experiences were not reported by our participants. This might be due to the very different setting on board of such navy vessels and consequently the differences in type of the tasks, workloads and stressors.

In the offshore petroleum industry, sleep problems were more pronounced in shift workers as compared to day workers, as shown in a systematic review [38]. In our study, these two groups did not significantly differ in mean PSQI score. Also, the only complain of the shift workers was having pain while sleeping. We therefore assume that this may be related to the unsimilar workplace setting. Offshore platforms are more stable than sailing vessels but around 100 people live and work on such platforms; this may induce more noise that can disturb sleep especially for shift workers while the others are working.

In the oil and gas offshore setting, both night shift and day workers reported impaired sleep quality in a qualitative study. Participants reported sleep disruption due to environmental stressors like noise of the platform, ergonomic requirement (e.g., quality of mattresses) and roommates [39]. These findings among offshore workers are not in line with ours as mentioned earlier it might be because of the differences of the workplace setting.

### Stress

Our study revealed that factors related to the nature of occupation and psychosocial work environment are perceived as the most important stressors on board of oil tankers. Routine inspections and long voyages without a day-off were pointed out by interviewees as inevitable stressors. Also, top-down hierarchical management structure and maltreatment of higher ranks towards other crew members were mentioned as psychosocial work-related stressors. Finally, family issues and loneliness were mentioned. Family issues (e.g., illness) were also found to be relevant stressors for seafarers in a review [3]. To minimize the adverse effects of loneliness and family issues, possibilities like easy access to the internet should be considered by the shipping companies.

A cross-sectional study demonstrated that during port stay, seafarers are engaged with a variety of tasks, and accumulation of such demanding tasks can cause more stress and strain on crew members. Also, one fifth of the studied population reported more stress during port stay [40]. Our findings about the stressors at sea are in line with the results of the mentioned cross-sectional study; it seems that heavy workloads during port stay, or load and discharge are the common points of both studies regarding stressful situations. Therefore, captains should give clear instructions regarding the next port activities to manage the workloads of the crew members. Also, interventions with more emphasis on spiritual growth (e.g., meditation in the frame of strong connection to higher powers) should be investigated. This might be one of the preventive actions suggested to be taken and investigated in terms of stress management in specific Asian workplace culture.

### Social communication

Participants of our study pointed out that lack of social communication has negative effects on both their work lives and private lives. Separation from family and society without good communication means might, together with the particularly disciplined culture at the workplace, have significant effects on their social lives on land. Separation from home, especially at times when the family is ill, has been introduced as one of the significant stressors among employees of offshore industries [40]. Inadequate communication with family and friends was mentioned as a main reason for seafarers’ job dissatisfaction that may even hinder retention in the profession [41]. Living and working in an isolated work environment for a long period of time without internet access has been considered a strong psychosocial stressor, reducing seafarers’ job satisfaction and health [42].

These findings imply that technology advancement and good communication means might considerably improve seafarers’ stress management, social life on land, job satisfaction, and professional retention.

## Conclusion

Sleep abnormalities and lack of communication should be considered as modifiable risk factors for seafarers’ psychosocial well-being at sea. The establishment of internal rules by shipping companies to tackle with such psychosocial challenges on board is highly recommended. In particular, providing good internet connection as a means of communication should be mandatory. This will likely help seafarers to sleep better and to control their stress level. Shipping companies should also encourage their employees to make a clear boundary between work and leisure time, something that would be made possible by operation department of the shipping company giving clear voyage instruction (e.g., next port of call, anchor time, sailing time) and a precise time plan for load and discharge.

### Limitations and strengths of the study

One limitation of this study is related to using self-administrative questionnaires where recall bias might have influenced the quantitative results. In addition, the quantitative part did not assess the additional dimensions of stress and communication that were revealed in the qualitative phase of our study. Instead, we concentrated on the aspect of sleep in the quantitative part because sleep disorders were a main problem of the participants; shipping company disagreed to send more questionnaires due to heavy workloads of the vessels.

One of the strengths of the study is that this is the first mixed method study that has addressed psychosocial challenges and quality of sleep among Asian seafarers. The presence of the main researcher on board during the qualitative phase of the study provided the opportunity to observe living and working conditions at sea closely. Depth data immersion was provided by such stay on board. Such observation also enabled the researcher to capitalize on opportunities to broaden and diversity the sample.

## Data Availability

The datasets generated and analyzed during the current study are not publicity available due to confidential policy of the shipping company.

## Abbreviations

VLCC: Very large crude carrier tankers
PSQI: Pittsburgh Sleep Quality Index
